# Changes in interoception before and after treatment in patients with alcohol use disorder

**DOI:** 10.1002/npr2.12491

**Published:** 2024-10-22

**Authors:** Chika Shimohara, Ariyuki Kagaya, Tomoyuki Akita, Ryotaro Tsukue, Atsushi Shimohara, Maro G. Machizawa, Shigeto Yamawaki, Junko Tanaka, Hitoshi Okamura

**Affiliations:** ^1^ Department of Psychosocial Rehabilitation, Graduate School of Biomedical and Health Sciences Hiroshima University Hiroshima Japan; ^2^ Senogawa Hospital Senogawa Medical Corporation Hiroshima Japan; ^3^ Yokogawa Ekimae Clinic Senogawa Medical Corporation Hiroshima Japan; ^4^ KONUMA Memorial Institute of Addiction and Mental Health Senogawa Hospital Hiroshima Japan; ^5^ Department of Epidemiology, Infectious Disease Control and Prevention, Graduate School of Biomedical and Health Sciences Hiroshima University Hiroshima Japan; ^6^ Center for Brain, Mind & KANSEI Sciences Research Hiroshima University Hiroshima Japan; ^7^ Xiberlinc Inc. Tokyo Japan

**Keywords:** alcohol use disorder, BPQ‐BAVSF, interoception, liver function

## Abstract

**Aims:**

To investigate the factors associated with interoception in patients with alcohol use disorder and determine whether treatment causes changes in their interoception.

**Methods:**

The Body Perception Questionnaire‐Body Awareness ultra‐short version Japanese version (BPQ‐BAVSF‐J) was used to measure interoception in 50 alcohol‐dependent participants (27 in the inpatient group and 23 in the outpatient group). The BPQ‐BAVSF‐J was administered and data on aspartate aminotransferase (AST), alanine aminotransferase (ALT), gamma‐glutamyl transpeptidase (γ‐GTP), mean corpuscular volume, platelet count, and Fib‐4 index were extracted at admission and immediately before discharge for the inpatient group and at the first outpatient visit and approximately 3 months after the visit for the outpatient group.

**Results:**

The mean age of the 50 participants was 51.0 ± 12.3 years. Significant associations were found between the BPQ‐BAVSF‐J and Fib‐4 index and AST. The BPQ‐BAVSF‐J score significantly decreased at discharge in the inpatient group. AST, ALT, γ‐GTP, and Fib‐4 index of liver function were also significantly lower at discharge. In contrast, in the outpatient group, there were no significant changes in the BPQ‐BAVSF‐J score, AST level, ALT level, γ‐GTP level, and Fib‐4 index between at the first outpatient visit and approximately 3 months after the visit.

**Conclusions:**

Interoception in patients with alcohol use disorder increased with worsening liver function and decreased with improvement in liver function owing to treatment. This suggests that the BPQ‐BAVSF‐J score, an easily accessible scale, may be used to detect early deterioration of liver function through regular administration.

## Abstract 2

Interoception in patients with alcohol use disorders increases with worsening liver function and decreases as liver function improves because of treatment. This suggests that the BPQ‐BAVSF‐J helps detect early deterioration of liver function through regular administration.

## Graphical abstract

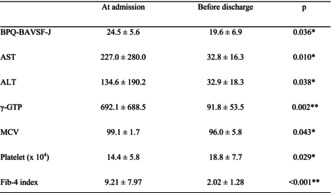

## 1 INTRODUCTION

The senses include exteroception, which is sensitivity to external stimuli; proprioception, which includes the sense of movement and equilibrium; and interoception, which is the awareness of the body's internal environment and its changes. The range of interoception is so broad that some researchers have recently argued that proprioception should also be included in it.^1^, ^2^ Interoception refers to the awareness of bodily signals that are not objectively known to others. Several previous studies have shown that physical sensations influence subsequent emotions and behavior.^3^, ^4^, ^5^, ^6^

Interoception is reportedly associated with psychiatric disorders, and changes in interoception may be affected in a wide range of psychiatric disorders, including depressive disorders,^7^ eating disorders,^8^, ^9^ obsessive– compulsive disorders,^10^, ^11^ substance use disorders,^12^ and posttraumatic stress disorder.^13^ Reports on interoception in patients with alcohol use disorder, such as the somatic marker hypothesis, suggest that an addict's awareness of their own body has some influence on the way they process emotions, such as cravings and decision‐making.^14^, ^15^ If the characteristics of interoception are linked to alcohol use disorder, then, interoception may serve as an indicator of early treatment and prevention of the disorder. However, to date, it remains unclear which factors are associated with interoception in patients with alcohol use disorder and how they change with treatment.

There are several methods for examining interoception. The heartbeat perception test, which counts one's heart rate and measures whether one can accurately perceive the heartbeat, is the most commonly used measurement method. There are also various methods, such as inserting a balloon into the gastrointestinal tract and changing the pressure to measure interoception other than the heartbeat, physiological testing methods by adjusting blood pressure and pulse rate, and electroencephalogram (EEG)‐heartbeat‐evoked potentials, that measure the relationship between the heartbeat and the amplitude of EEG.^6^, ^7^, ^16^ The Body Perception Questionnaire was originally developed by Porges.^17^ It is a 122‐item test consisting of five subscales: body awareness (BA), stress response, autonomic response, coping with stress, and history of stress. Furthermore, the BPQ‐BA very short form (BPQ‐BAVSF) was created by reducing the number of items to 12.^18^

In this study, the BPQ‐BAVSF was used to measure interoception. We aimed to investigate the factors associated with interoception in patients with alcohol use disorder and whether treatment causes changes in their interoception.

## 2 METHODS

### 2.1 Participants

Of those who visited Senogawa Hospital or Yokogawa Ekimae Clinic between September 1, 2021, and March 31, 2023, and initiated inpatient or outpatient treatment, 50 individuals had a diagnosis of alcohol use disorder according to the Diagnostic and Statistical Manual of Mental Disorders, Fifth Edition, and who provided written consent for the study. Of the 50 participants, 27 received inpatient treatment (inpatient group), and 23 received outpatient treatment (outpatient group).

### 2.2 Measures

#### 2.2.1 Basic information

Age, sex, history of psychiatric hospitalization, Alcohol Use Disorders Identification Test (AUDIT), AUDIT Consumption (AUDIT‐C), blood data (aspartate aminotransferase [AST], alanine aminotransferase [ALT], gamma‐glutamyl transpeptidase [γ‐GTP], mean red blood cell volume, platelets, Fib‐4 index), systolic and diastolic blood pressure, and pulse rate were extracted from the medical records of the participants.

#### 2.2.2 AUDIT and AUDIT‐C

The AUDIT and AUDIT‐C are screening tests for problem drinking. Developed by the World Health Organization, the AUDIT consists of 10 questions.^19^ Each item is scored from 0 to 4 points, with the total score ranging from 0 to 40 points; higher scores indicate more problematic drinking. Problem drinkers were rated from 8 to 14 points, and those scoring ≥15 points were suspected of having addiction problems. A score of ≥6 points for Japanese men and ≥4 points for Japanese women corresponded to an AUDIT score of ≥8 points (problem drinkers), indicating the need for therapeutic intervention. The AUDIT‐C is a modified version of the AUDIT, consisting of a three‐item screen for hazardous alcohol use.^20^ It focuses on alcohol consumption and serves as a screening test for problem drinkers. Each item is scored on a scale of 0–4 points, resulting in a total score of 0–12 points. For Japanese men, a score of ≥6 points, and for Japanese women, a score of ≥8 points on the AUDIT‐C indicates the need for therapeutic intervention. In this study, as the AUDIT and AUDIT‐C measure the degree of problematic drinking, measurements were taken only at the time of admission and at the first outpatient visit.

#### 2.2.3 Japanese version of the BPQ‐BAVSF (BPQ‐BAVSF‐J)

The BPQ‐BAVSF‐J consists of 12 questions regarding thirst, breathing, body swelling, muscle tension, feelings of swelling, goose bumps, gastrointestinal pain, abdominal fullness, lip tremors, feelings of skin inversion, feelings of spitting, and heartbeat. Each question was rated on a scale of 1–5, resulting in a minimum score of 12 and a maximum score of 60, with higher scores indicating greater interoception. The BPQ‐BAVSF‐J used in the current study has been confirmed to be reliable and valid.^18^

### 2.3 Evaluation method

For the inpatient group, the BPQ‐BAVSF‐J was administered at admission and immediately before discharge, and data on AST, ALT, γ‐GTP, mean corpuscular volume (MCV), platelet count, and Fib‐4 index were extracted at admission and immediately before discharge. For the outpatient group, the BPQ‐BAVSF‐J was administered at the first outpatient visit and approximately 3 months after the visit, and data on AST, ALT, γ‐GTP, MCV, platelet count, and Fib‐4 index were extracted at the first outpatient visit and approximately 3 months after the visit. The second evaluation was conducted after 3 months to align with the expected length of hospital stay for the inpatient group and to match the typical interval for outpatient follow‐up evaluations, which are often scheduled approximately 3 months after the initial visit.

### 2.4 Statistical analysis

Continuous variables are reported as means ± standard deviations. Comparisons between the outpatient and inpatient groups were made using Student's *t*‐test, while changes in each variable were assessed using paired *t*‐tests. The relationship between the BPQ‐BAVSF‐J and each indicator and the relationship between changes in the BPQ‐BAVSF‐J and changes in the AST level, ALT level, γ‐GTP level, and Fib‐4 index were assessed using Pearson's correlation coefficient.

Statistical analyses were performed using JMP Pro 16 software (SAS Institute Japan Co., Ltd., Tokyo, Japan). Statistical significance was set at *p* < 0.05.

### 2.5 Ethical considerations

The Senogawa Medical Corporation Ethics Committee approved this study (approval number: R03‐05‐06). Written informed consent was obtained from all participants before the study procedure. All study activities were performed in accordance with the Declaration of Helsinki and relevant guidelines and regulations in Japan.

## 3 RESULTS

### 3.1 Associations between the BPQ‐BAVSF‐J and each indicator at admission and the first outpatient visit

The mean age of the 50 participants was 51.0 ± 12.3 years, comprising 39 men and 11 women. The means and standard deviations of the BPQ‐BAVSF‐J scores at admission and the first outpatient visit are shown in Table 1. Table 2 compares data between the inpatient and outpatient groups. Age and BPQ‐BAVSF‐J were higher in the inpatient group, whereas AUDIT, AUDIT‐C, AST/ALT, MCV, and platelet counts showed no significant differences between the groups. Significant correlations were found between BPQ‐BAVSF‐J scores and both the Fib‐4 index and AST levels (Table 3).

**TABLE 1:** Baseline data of participants.

	Mean ± standard deviation
Age	51.0 ± 12.3
AUDIT	25.8 ± 6.1
AUDIT‐C	10.0 ± 2.6
BPQ‐BAVSF‐J	22.1 ± 6.1
AST	127.2 ± 189.7
ALT	76.8 ± 122.9
AST/ALT	1.76 ± 0.81
γ‐GTP	404.2 ± 518.2
MCV	98.1 ± 7.2
Platelet (x 10^4^)	19.7 ± 9.1
Fib‐4 index	5.46 ± 6.39
Systolic blood pressure	128.5 ± 19.1
Diastolic blood pressure	83.0 ± 12.0
Pulse	88.0 ± 16.4

Abbreviations: ALT, alanine aminotransferase; AST, aspartate aminotransferase; AUDIT, Alcohol Use Disorders Identification Test; AUDIT‐C, AUDIT Consumption; BPQ‐BAVSF‐J, Body Perception Questionnaire‐Body Awareness Ultra‐Short Version Japanese version; MCV, mean corpuscular volume; γ‐GTP, gamma‐glutamyl transpeptidase.

**TABLE 2:** Comparison between inpatient at admission and outpatient at first outpatient visit.

	Outpatient (*n* = 23)	Inpatient (*n* = 27)	*p*
Age	47.0 ± 11.0	54.4 ± 12.4	0.032*
AUDIT	25.1 ± 6.1	26.4 ± 6.2	0.466
AUDIT‐C	9.4 ± 3.2	10.6 ± 1.8	0.102
BPQ‐BAVSF‐J	20.0 ± 6.0	23.9 ± 5.8	0.020*
AST	62.8 ± 67.4	182.0 ± 239.2	0.025*
ALT	37.4 ± 29.1	110.3 ± 158.8	0.035*
AST/ALT	1.54 ± 0.50	1.95 ± 0.98	0.081
Γ‐GTP	233.5 ± 327.7	549.6 ± 606.2	0.030*
MCV	98.1 ± 7.5	98.0 ± 7.1	0.972
Platelet	22.1 ± 9.5	17.7 ± 8.3	0.091
Fib‐4 index	2.95 ± 3.43	7.50 ± 7.50	0.011*

*Note*: *p*, *t*‐test.

Abbreviations: ALT, alanine aminotransferase; AST, aspartate aminotransferase; AUDIT, Alcohol Use Disorders Identification Test; AUDIT‐C, AUDIT Consumption; BPQ‐BAVSF‐J, Body Perception Questionnaire‐Body Awareness Ultra‐Short Version Japanese version; MCV, mean corpuscular volume; γ‐GTP, gamma‐glutamyl transpeptidase.

* *p* < 0.05.

**TABLE 3:** Relationship between the BPQ‐BAVSF‐J and each indicator.

	*r*	*p*
Age	−0.05	0.737
AUDIT	0.24	0.091
AST	0.28	0.046*
ALT	0.22	0.133
AST/ALT	0.18	0.200
γ‐GTP	0.12	0.391
MCV	−0.14	0.327
Platelet	−0.08	0.584
Fib‐4 index	0.30	0.038*
Systolic blood pressure	0.01	0.947
Diastolic blood pressure	−0.29	0.058
Pulse	0.19	0.216

*Note*: *r*, Pearson's correlation coefficient.

Abbreviations: ALT, alanine aminotransferase; AST, aspartate aminotransferase; AUDIT, Alcohol Use Disorders Identification Test; AUDIT‐C, AUDIT Consumption; BPQ‐BAVSF‐J, Body Perception Questionnaire‐Body Awareness Ultra‐Short Version Japanese version; MCV, mean corpuscular volume; γ‐GTP, gamma‐glutamyl transpeptidase.

* *p* < 0.05.

### 3.2 Change over time for each indicator

In the inpatient group, BPQ‐BAVSF‐J decreased significantly from 24.5 ± 5.6 at admission to 19.6 ± 6.9 before discharge. Significant changes were also observed for AST level, ALT level, γ‐GTP level, MCV, platelet count, and Fib‐4 index at discharge (Table 4). In contrast, in the outpatient group, there were no significant changes in BPQ‐BAVSF‐J scores, AST level, ALT level, γ‐GTP level, MCV, platelet count, and Fib‐4 index (Table 5).

**TABLE 4:** Comparison of indicators between at admission and before discharge in the inpatient group.

	At admission	Before discharge	*p*
BPQ‐BAVSF‐J	24.5 ± 5.6	19.6 ± 6.9	0.036*
AST	227.0 ± 280.0	32.8 ± 16.3	0.010*
ALT	134.6 ± 190.2	32.9 ± 18.3	0.038*
γ‐GTP	692.1 ± 688.5	91.8 ± 53.5	0.002**
MCV	99.1 ± 1.7	96.0 ± 5.8	0.043*
Platelet (×10^4^)	14.4 ± 5.8	18.8 ± 7.7	0.029*
Fib‐4 index	9.21 ± 7.97	2.02 ± 1.28	<0.001**

*Note*: *p*, paired *t*‐test.

Abbreviations: ALT, alanine aminotransferase; AST, aspartate aminotransferase; AUDIT, Alcohol Use Disorders Identification Test; AUDIT‐C, AUDIT Consumption; BPQ‐BAVSF‐J, Body Perception Questionnaire‐Body Awareness Ultra‐Short Version Japanese version; MCV, mean corpuscular volume; γ‐GTP, gamma‐glutamyl transpeptidase.

*p < 0.05, **p < 0.01.

**TABLE 5:** Comparison of indicators between at the first outpatient visit and approximately 3 months after the visit in the outpatient group.

	At the first outpatient visit	Approximately 3 months after the visit	*p*
BPQ‐BAVSF‐J	18.4 ± 3.2	18.7 ± 3.5	0.900
AST	63.0 ± 77.2	59.4 ± 36.9	0.853
ALT	38.7 ± 35.4	48.3 ± 42.1	0.631
γ‐GTP	374.0 ± 398.0	273.0 ± 325.2	0.119
MCV	99.3 ± 5.5	99.7 ± 7.8	0.774
Platelet (×10^4^)	22.7 ± 11.6	20.3 ± 7.2	0.613
Fib‐4 index	2.83 ± 2.97	2.73 ± 2.17	0.859

Abbreviations: ALT, alanine aminotransferase; AST, aspartate aminotransferase; AUDIT, Alcohol Use Disorders Identification Test; AUDIT‐C, AUDIT Consumption; BPQ‐BAVSF‐J, Body Perception Questionnaire‐Body Awareness Ultra‐Short Version Japanese version; MCV, mean corpuscular volume; γ‐GTP, gamma‐glutamyl transpeptidase.

*p*, paired *t*‐test.

### 3.3 Relationship between changes in BPQ‐BAVSF‐J and changes in various indicators of liver function

The change in BPQ‐BAVSF‐J and the change in AST level, ALT level, γ‐GTP level, MCV, platelet count, and Fib‐4 index showed no significant association (Table 6).

**TABLE 6:** Relationship between changes in BPQ‐BAVSF‐J and changes in indices of liver function.

	*r*	*p*
BPQ‐BAVSF‐J change − AST change	0.31	0.131
BPQ‐BAVSF‐J change − ALT change	0.33	0.100
BPQ‐BAVSF‐J change − ALT/AST change	−0.04	0.823
BPQ‐BAVSF‐J change − γ‐GTP change	0.05	0.779
BPQ‐BAVSF‐J change − MCV change	−0.13	0.526
BPQ‐BAVSF‐J change − platelet change	0.03	0.867
BPQ‐BAVSF‐J change − Fib‐4 index change	0.36	0.072

*Note*: *r*, Pearson's correlation coefficient.

Abbreviations: ALT, alanine aminotransferase; AST, aspartate aminotransferase; AUDIT, Alcohol Use Disorders Identification Test; AUDIT‐C, AUDIT Consumption; BPQ‐BAVSF‐J, Body Perception Questionnaire‐Body Awareness Ultra‐Short Version Japanese version; MCV, mean corpuscular volume; γ‐GTP, gamma‐glutamyl transpeptidase.

## 4 DISCUSSION

The participants in this study were patients with alcohol use disorder and problematic drinking, as indicated by their AUDIT and AUDIT‐C scores. The higher values for age, AST level, ALT level, γ‐GTP level, and the Fib‐4 index in the inpatient group may reflect the advanced progression of alcohol dependence and associated liver function deterioration. The lack of significant difference in AUDIT and AUDIT‐C scores between the two groups suggests that subjective symptoms of dependence may be similar regardless of inpatient or outpatient status. In contrast, the significant difference in BPQ‐BAVSF‐J scores between the groups indicates that patients might experience heightened sensory discomfort, such as visceral and cutaneous sensations, which could influence their decision to seek hospitalization. Further research is needed to explore these sensory experiences in more detail.

Regarding the factors associated with the BPQ‐BAVSF‐J at admission and the first outpatient visit, there was no significant association with age, indicating that interoception is not affected by age in alcohol use disorder. There was no significant association between the BPQ‐BAVSF‐J and AUDIT scores, indicating that the BPQ‐BAVSF‐J is not an indicator of the severity of problem drinking. In contrast, an association was observed between the BPQ‐BAVSF‐J, AST, and Fib‐4 index scores. AST is an indicator of hepatocellular damage, and high AST levels may indicate acute hepatitis, cirrhosis, or liver cancer. The Fib‐4 index is an indicator of hepatic fibrosis, and the progression of fibrosis can lead to liver cirrhosis. There are three main categories of alcohol‐induced liver injury: fatty liver, alcoholic hepatitis, and alcoholic cirrhosis, among which cirrhosis is the most serious and life‐threatening. Fibrosis, in particular, appears when the condition progresses from fatty liver to alcoholic hepatitis, during which the symptoms of fever, jaundice, and abdominal pain appear.^21^ The results of the present study show that when liver dysfunction becomes so severe that the Fib‐4 index and AST become abnormal, the interoception indicated by the BPQ‐BAVSF‐J becomes high. However, further research is needed to elucidate the mechanism of why interoception is elevated in patients with alcohol use disorder as liver dysfunction worsens.

In the inpatient group, the BPQ‐BAVSF‐J scores significantly decreased with treatment, and liver function improved simultaneously. In contrast, in the outpatient group, BPQ‐BAVSF‐J scores remained low after 3 months of treatment, and there was no change in liver function. This may suggest that the lack of progress in treating alcohol use disorder during the 3 months of outpatient treatment resulted in no change in liver function indices and, accordingly, no change in interoception. The present results suggest that interoception may have decreased with improvement in liver dysfunction. However, since no significant correlation was found between the amount of change in BPQ‐BAVSF‐J scores and the amount of change in indices of liver function, the degree of improvement in liver dysfunction may not be associated with the degree of decrease in interoception. While it has been shown that patients with alcohol use disorder have less accurate interoception and are less likely to notice physical signs,^22^ it is not clear from this study why interoception decreased as liver function improved, and further research is needed. However, the simplicity of the BPQ‐BAVSF‐J and its ease of use in clinical practice suggest that regular administration of this scale may help detect early deterioration of liver function.

The limitations of this study include the following. First, the sample size was not large enough to examine inpatients and outpatients in detail. Second, other factors that may influence interoception have not been adequately examined. Third, the association with other physical diseases, including alcoholic cirrhosis, could not be examined. Finally, as the BPQ‐BAVSF is a self‐report questionnaire measuring subjective interoceptive awareness, it might not accurately reflect the objective interoception.

In conclusion, this study showed that interoception in patients with alcohol use disorder increased with worsening liver function and decreased with improvement in liver function. The BPQ‐BAVSF‐J score, an easily accessible scale, may help detect early deterioration of liver function through regular administration.

## AUTHOR CONTRIBUTIONS

C.S., A.K., and H.O. contributed to the conception and design of this study and wrote the manuscript. C.S., R.T., and A.S. contributed to the data acquisition. T.A., M.M., and J.T. performed the data analysis. C.S., S.Y., and H.O. interpreted the data. All the authors contributed to and approved the final version of the manuscript.

## FUNDING INFORMATION

The authors received no funding for this research.

## CONFLICT OF INTEREST STATEMENT

The authors declare no conflict of interest.

## ETHICS STATEMENT

Approval of the research protocol by an Institutional Review Board: The Senogawa Medical Corporation Ethics Committee approved this study (approval number: R03‐05‐06). All study activities were performed in accordance with the Declaration of Helsinki and relevant guidelines and regulations in Japan.

Informed consent: Written informed consent was obtained from all participants before the study procedure.

Registry and the registration no. of the study/trial: N/A.

Animal studies: N/A.

## Supporting information


Appendix S1.

## Data Availability

All relevant data are provided in the manuscript. The raw data of this study are available in the Supporting Information.
